# Comparative dataset on growth, development and reproductive performance of copepod (*Cyclops* sp.) fed with different microalgae diet

**DOI:** 10.1016/j.dib.2024.110863

**Published:** 2024-08-29

**Authors:** Sabiha Zaman Usha, Mahima Ranjan Acharjee, Subeda Newase, Trina Das, Sifatun Nur, Mohammad Ekramul Haque, Md. Shakib Hossain, Helena Khatoon

**Affiliations:** Department of Aquaculture, Chattogram Veterinary and Animal Sciences University, Chattogram 4225, Bangladesh

**Keywords:** Specific growth rate, Survival rate, Development time, Hatching rate, Lifespan

## Abstract

The effects of growth and reproduction on the marine *Cyclops* sp. were investigated using three microalgae as diets. The development period of *Cyclops* sp. was evaluated at 10^6^ cells/ml in 15ppt salinity to identify the stationary phase. The survival rate of marine *Cyclops* from nauplius to adult differed according to the microalgal diet. The results showed that the shortest time (14 days) and highest survival (17.6 ± 0.131 %) for *Cyclops* sp. was achieved with those fed with *Nannochloropsis* sp. Whereas, it took longest time (37 days) and lowest survival rate (6.40 ± 0.035 %) when fed C*hlamydomonas* sp. The developmental period from naupli (I - VI) (6.91 ± 0.453 days), copepodite (I - VI) (11.4 ± 0.311days) and naupli to adult (20 ± 1.08 days) appeared significantly longer when fed with *Nannochloropsis* sp. compared to other treatments. The daily mean naupli production of adult females over 7 days was significantly higher (*p* ˂ 0.05) in *Nannochloropsis* sp. compared with *Chlamydomonas* sp. and *Gonyostomum* sp. On the 25th day of *Nannochloropsis* sp. treatment, 99 % of the mature females died. Production (naupli, copepodite adult male and adult female) was significantly higher (*p* ˂ 0.05) in *Nannochloropsis* sp. than in other microalgal diets. On the fifteenth day, *Nannochloropsis* sp. showed a significantly higher (*p* ˂ 0.05) specific growth rate than other microalgal diets. *Nannochloropsis* sp. had the highest nauplius survival rate on the sixth day compared to other microalgal diets. With *Nannochloropsis* sp*.*, the species has a higher hatching rate, and in *Chlamydomonas* sp. hatching occurs earlier. The average lifespan for *Nannochloropsis* sp. was 46 days, for *Chlamydomonas* sp. it was 37 days, and for *Gonyostomum* sp. it was 32 days.

Specifications Table

**Table:** 

Subject	Food Science, Aquatic Science
Specific subject area	Effect of different microalgae as diets on the growth and reproduction of marine zooplankton *Cyclops* sp.
Data format	Raw and analyzed
Type of data	Table and graph
Data collection	Population growth and reproduction was determining daily from each treatment with five replicates in order to calculate and recorded separately. The copepod was examined at regular intervals (every 2 h) for the hatching of naupli. Once the naupli released, the adult female was carefully removed from the test tube and the naupli were counted under the microscope with Sedgewick-Rafter chamber. In this experiment, the time taken for eggs to hatch from spawning (embryonic development), the development of nauplius stages (NI – NVI) and copepodite stages (CI – CVI) were noted and generation time (egg to egg) was also recorded. Few naupli from each treatment were cultured separately until they spawn to determine the development time, survival and sex ratio. The acquired data were further analyzed through MS Excel.
Data source location	Disease and Microbiology lab, Department of Aquaculture, Faculty of Fisheries, Chattogram Veterinary and Animal Sciences University, Khulshi-4225, Chattogram, Bangladesh
Data accessibility	Data are available with this article and also at Repository name: Mendeley Data Data identification number: 10.17632/h5rkyyc6tr.1 Direct URL to data: https://data.mendeley.com/datasets/h5rkyyc6tr/1

## 1 Value of the Data

• The data will contribute to the selection of potential microalgae culture for marine *Cyclops* sp.
• Data on population growth, developmental stages, and survival, will be useful in selecting microalgae with the best potential as live feed source in the mass production of copepod for aquaculture application.
• Data on growth (population growth and survival rates) and reproductive parameters (developmental stages, sex ratio, fecundity, and egg hatchability) are important basic information in the study of copepod biology as well as guide for further investigations or applications in copepod ecology and aquaculture application.

## 2 Background

Zooplankton is a marine microorganism that forms primary and secondary linkages in the food webs of all aquatic habitats [2,3]. Copepods are commonly used as live feed in the aquaculture sector, notably for marine fish along with crustacean larvae [4]. Marine copepods are commercially cultivable species their growth and survival have been impacted by different feeding habit [[5], [6], [7]]. Food quality and quantity are also major factors influencing copepod growth and fecundity. Copepods require algal feeding, which promotes the growth of marine *Cyclops* sp*.* The present study investigated the growth and population of marine *Cyclops* sp. fed with different microalgae. Although certain marine copepod species have been successfully grown in the past, there are still very few species that have been recorded. The use of *Cyclops* as live food has various benefits, including enhanced fish and crustacean larval survival, accelerated larval growth, and provision of essential nutrients.

There is currently no documentation on the culture of this *Cyclops* sp*.* or their parameters when given microalgal diets. The main issue with using copepods as live feed in the aquaculture sector is inconsistent production due to poor culture procedures and a lack of information about their optimal diets [8].

## 3 Data Description

Comparison on three microalgae diets on copepods growth, development and reproductive performance. Copepod survival rates and specific growth rate differed between different microalgae (*p* < 0.05, Table 1). Copepods fed with *Nannochloropsis* sp*.* (17.6 ± 0.131 %) had a highest survival rate among the diets presented (*Gonyostomum* sp. at 14.40 ± 0.035 % and *Chlamydomonas* sp*. at* 6.4 ± 0.035 %) [1].

**Table 1:** Effects of *Cyclops* sp. survival rate (%) and specific growth rate fed with different microalgae. All values are mean ± SD (*n* = 5). Different small letters indicate significant differences between treatments (*p* < 0.05).

Treatments	Survival rate (%)	Specific growth rate
*Nannochloropsis* sp.	17.60 ± 0.131^a^	0.09 ± 0.004^a^
*Gonyostomum* sp.	14.40 ± 0.035^b^	0.08 ± 0.005^b^
*Chlamydomonas* sp.	6.40 ± 0.035^c^	0.07 ± 0.007^c^

The total population growth rate of *Cyclops* sp. per dietary treatment after 25 days of culture varied based on food type (*p* < 0.05, Table 2). Copepods fed *Nannochloropsis* sp. achieved highest number of naupli, copepodite and adult mane. On the other hand, Copepods fed *Gonyostomum* sp. grow more than *Chlamydomonas* sp. (*p* < 0.05). Table 2 shows the population growth rate of copepods fed multiple microalgae diets.

**Table 2:** Developmental stages (no. of individuals) and sex ratio (M : F) fed with three different microalgae. All values are mean ± SD (*n* = 5). Significant differences between treatments are indicated by small letters (*p* < 0.05). All of the data represents the average of individual's number except sex ratio.

Treatments	Naupli	Copepodite	Adult Male	Adult Female	Sex ratio
*Nannochloropsis* sp.	28.8 ± 3.03^a^	22.8 ± 2.68^a^	18.4 ± 3.85^a^	4.4 ± 2.61^a^	77 ± 15.9^a^
*Gonyostomum* sp.	20.8 ± 2.28^b^	15.6 ± 2.97^b^	11.6 ± 1.67^b^	3.6 ± 1.67^a^	48.6 ± 6.96^b^
*Chlamydomonas* sp.	14.4 ± 2.97^c^	10.8 ± 2.28^c^	8 ± 3.74^b^	2.4 ± 1.67^a^	35.5 ± 15.5^b^

The sex ratios of marine *Cyclops* sp. differ significantly (*p* < 0.05, Table 2). Copepods fed with *Nannochloropsis* sp. had the highest sex ratio where males were dominant (77 ± 15.9 %; Table 2), followed by copepods fed with *Gonyostomum* sp. (48.6 ± 6.96 %), and *Chlamydomonas* sp. (33.5 ± 15.5 %).

**Fig. 1:**
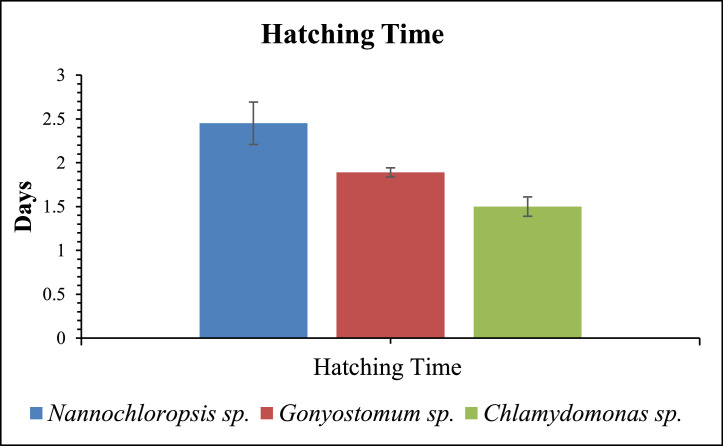
Hatching time of copepods fed with different microalgal diets.

Different microalgae significantly impacted the Copepod egg hatching time (*p* < 0.05, Fig. 1). Copepods fed with fresh *Chlamydomonas* sp. showed the shortest egg-hatching time (1.5 ± 0.11 days). Whereas, copepods fed with *Nannochloropsis* sp. (2.45 ± 0.242 days) had the longest egg hatching time.

**Fig. 2:**
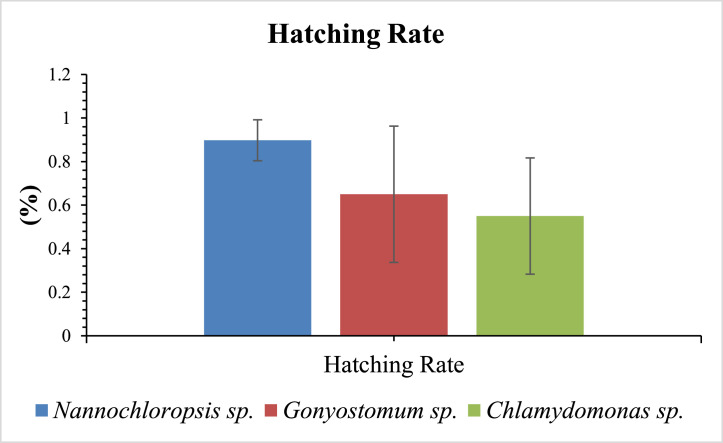
Hatching rate of copepods fed with different microalgal diets.

According to the graph presented there was no significance in terms of hatching rate of female copepods by algal diet type (*p* < 0.05, Fig. 2). Copepods fed with *Nannochloropsis* sp., *Gonyostomum* sp., *Chlamydomonas* sp. showed similar hatching rate (1.5 ± 0.11 %).

**Fig. 3:**
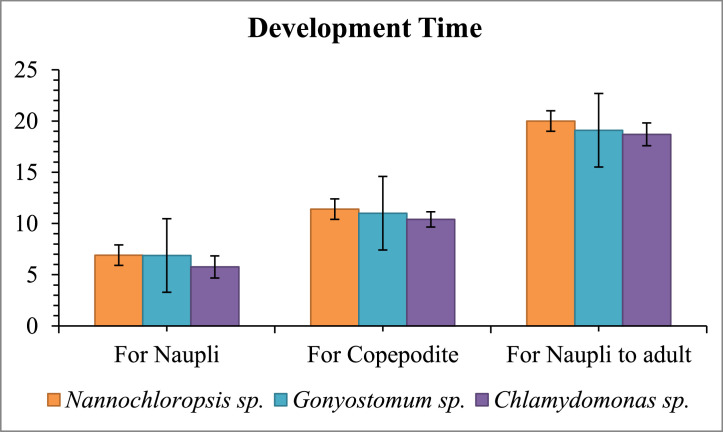
Development time of naupli to adult fed with different microalgae.

Copepods fed *Chlamydomonas* sp. exhibited the development time (11.38 ± 0.139 days) compared to other dietary regimens (*p* < 0.05). Copepodite maturation periods did not differ substantially between food types (*p* = 0.091, Fig. 3).

Copepods fed *Nannochloropsis* sp. (20 ± 1.08 days) had a longer development period from naupli to gravid female than those fed *Chlamydomonas* sp. (18.7 ± 1.11 days) (Fig. 3).

Table 3 showed the lifespans of female marine *Cyclops* sp. under various dietary treatments. Copepods fed on *Nannochloropsis* sp. had the highest lifespan (35.2 ± 2.39 days). while those fed with *Chlamydomonas* sp. had the shortest at 25.0 ± 2.55 days (Table 3).

Copepods produced the most spawns in their lives when fed *Nannochloropsis* sp*.* (4.2 ± 0.836). Different diets had a significant impact on the number of spawns in a lifetime (*p* < 0.05). Copepods fed with *Chlamydomonas* sp. diets had the lowest spawning rates (1.8 ± 0.447; Table 3).

The number of offspring produced per egg sac per spawn varied based on diet type, where those fed with *Nannochloropsis* sp*.* achieved the highest (6.4 ± 1.14), while those fed with *Chlamydomonas* sp*.* produced the fewest among diets (Table 3).

**Table 3:** Effects of reproductive performance of *Cyclops* sp. fed with different microalgae.

Treatments	Mean lifespan of copepods (Days)	No. of spawning/lifespan	Total offspring/egg sacs	Total offspring/female
*Nannochloropsis* sp.	35.2 ± 2.39^a^	4.2 ± 0.837^a^	6.4 ± 1.14^a^	28.8 ± 3.03^a^
*Gonyostomum* sp.	29.7 ± 1.57^b^	2.6 ± 0.548^b^	5 ± 0.707^ab^	20.8 ± 2.28^b^
*Chlamydomonas* sp.	25.0 ± 2.55^c^	1.8 ± 0.447^b^	4.2 ± 0.837^b^	14.4 ± 2.97^c^

## 4 Experimental Design, Materials and Methods

### 4.1 *Collection of zooplankton cyclops* sp*. and microalgal strains*

Pure strain of *Nannochloropsis* sp., *Gonyostomum* sp., and *Chlamydomonas* sp. and *Cyclops* sp. were collected from live feed research corner, Chattogram Veterinary and Animal Sciences University to conduct the experiment.

### 4.2 Stock culture

In stock culture, isolated species were transferred to a 1 L beaker filled with filtered seawater. During culture, 24-h aeration was provided; *Cyclops* sp*.* was fed with mixed algae at a concentration of 2 × 10^6^cells/ml for once in daily. The culture was then carried out continuously for numerous generations to ensure a mono-species stock. Additionally, wastes were siphoned and the expelled culture water was exchanged with filtered fresh seawater on daily basis.

### 4.3 Maintenance of microalgae culture

The purified samples were cultivated in Conway culture medium with 15 ppt salinity. The stock was scaled up and then mass-cultivated for *Cyclops* sp*.* diets.

### 4.4 Experimental design

The effects of microalgae diets on the reproductive development of *Cyclops* sp*.* were studied using three different microalgae *Nannochloropsis* sp., *Gonyostomum* sp., and *Chlamydomonas* sp. at 15 ppt salinity in the 8-hour dark and 16-h light on 24 h photoperiod. Throughout the experimental period copepod was measured and counted on daily basis under the microscope (Optika, Italy). These studies were carried out under controlled environment, with five replicates. The experiment was conducted in a petri dish with one gravid copepod and 40 ml of filtered autoclave seawater with three different microalgae diet.

The experiment starts with one adult female from the first breeding with egg sacs. They were given *Nannochloropsis* sp., *Gonyostomum* sp., and *Chlamydomonas* sp. with concentrations of 10^6^cells/ml maintained throughout the experiment. In the experiment, algal cells were supplied and counted daily using hemocytometer under the microscope (Optika, Italy) to maintain the same feed content. Cells were counted in the two chambers of the hemocytometer under a magnification of 40×. The following formula was used for the cell density calculation [9]:

$$\text{Cell}\;\text{count}\;\text{calculation}\;\left(\text{cell}/\text{mL}\right)\;\text{for}\;25\;\text{squares}\;=\frac{\text{Total}\;\text{number}\;\text{of}\;\text{cells}\;\text{counted}}{50\times 4}\times 10^{6}$$

In this equation, 50 stood for the 50 squares within each of the two hemocytometer chambers, and 4 × 10^−6^ for the volume of samples spread among the tiny square areas, which was equal to 0.004 mm^3^ (0.2 mm × 0.2 mm × 0.1 mm), expressed in cm^3^ (mL).

All replicates were monitored at 6-hour intervals to determine hatching and the lifespan. After the female copepods released their eggs, they were transferred to a different petri dish. A similar method was followed until the female produced more egg sacs.

### 4.5 Determination of population and individual growth

The experimental conditions were maintained constant for 25 days, with manual shaking every 2times per day. Each feeding treatment was replicated five times to identify the copepod population. Samples were collected from each treatment daily with replicates to calculate and record copepod development phases (naupli, copepodite, and adult) as well as copepod density.

Specific growth rate was calculated from the density data by using the following equation [10]:

R=[[lnNe⁡−lnNi]÷t]

Where, t is cultured days, Ni are the initial density of copepods and Ne is the end density of copepods.

### 4.6 Determination of survival rate

Survival rate of copepod was calculated by using the following equation [11]:

$$\begin{array}{ccc} \text{Survival}\;\text{rate} & = & (\text{total}\;\text{number}\;\text{of}\;\text{five}\;\text{samples}\;\text{taken}\;\text{once}\;\text{in}\;\text{two}\;\text{days}\\ & & ÷\;\text{total}\;\text{number}\;\text{of}\;\text{initial}\;\text{copepod}\;\text{density}\;\text{in}\;\text{petri}\;\text{dish})\times 100\% \end{array}$$

### 4.7 Determination of reproductive performance

#### 4.7.1 Hatching time and hatching rate

The eggs were monitored at 6-hour intervals to record the newly hatched naupli over a 24-h duration. Following a period of 48 h since hatching, the unhatched egg was tallied and documented. Equation use to calculate the hatching rate is as follows [11]:

Hatchingrate=[1−(numberofunhatchedeggs÷numberoftotaleggs)]

#### 4.7.2 Development time

The growth stages of copepods were observed under a microscope to determine their maturation period (from Nauplius to Copepodite, Copepodite to adult, and production of offspring from the nauplii survived from the 1st generation).

#### 4.7.3 Off spring production, spawning and lifespan

After spawning, each male-female couple was transferred to a new beaker to allow for better observation of future spawning. Offspring productions were calculated using the average of triplicate pairs of cyclops sp. in each treatment.

#### 4.7.4 Sex ratio

At the end of the experiment, the sex ratio was calculated by counting all male and female adults separately. Adult copepods were counted using a dissecting microscope (Nikon E600).

### 4.8 Quality assurance and quality control

Each instrument was calibrated by the applicable standard operating procedures (SOPs) and manufacturer instructions. Balance and other measuring equipment were regularly calibrated. Before starting the experiment, all equipment was checked properly. The tests that were undertaken produced consistent results. The experimental apparatus was cleaned with chlorine and sterilized in an autoclave before and after use.

### 4.9 Statistical analysis

Microsoft Excel was used to calculate the mean and standard deviation. All data were tested using a one-way analysis of variance (ANOVA). Statistical analyses were carried out using the IBM SPSS (v. 26.0) software. Significant differences between groups were determined at a *p*-value < 0.05.

## Limitations

Not applicable.

## CRediT Author Statement

**Sabiha Zaman Usha**- Conceptualization, data collection and drafting the manuscript; **Mahima Ranjan Acharjee**- review and editing; **Sifatun Nur, Subeda Newase, Trina Das**- data collection and formatting manuscript; **Mohammad Ekramul Haque** - data analysis, **Md. Shakib Hossain**- data collection; **Helena Khatoon**- Conceptualization, supervision, validation, review, editing, and submission.

## Ethical Statement

These data were collected complying ARRIVE guidelines. Ethical approval is not a prerequisite of starting the data collection procedure for microalgae and zooplankton.

## Data Availability

Comparative dataset on growth, development and reproductive performance of copepod (Cyclops sp.) fed with different microalgae diet (Original data) (Mendeley Data).
